# Conditional deletion of *Wntless* in granulosa cells causes impaired corpora lutea formation and subfertility

**DOI:** 10.18632/aging.202222

**Published:** 2020-12-03

**Authors:** Jinmei Cheng, Yinchuan Li, Yan Zhang, Xiuxia Wang, Fei Sun, Yixun Liu

**Affiliations:** 1 Institute of Reproductive Medicine, School of Medicine, Nantong University, Nantong 226001, China; 2 Key Laboratory of Fertility Preservation and Maintenance of Ministry of Education, Ningxia Medical University, Ningxia 751400, China; 3 State Key Laboratory of Stem Cell and Reproductive Biology, Institute of Zoology, Chinese Academy of Sciences, Beijing 100101, China

**Keywords:** WNTLESS, WNT signaling, ovary, granulosa cells, corpora lutea

## Abstract

WNT proteins are widely expressed in the murine ovaries. WNTLESS is a regulator essential for all WNTs secretion. However, the complexity and overlapping expression of WNT signaling cascades have prevented researchers from elucidating their function in the ovary. Therefore, to determine the overall effect of WNT on ovarian development, we depleted the *Wntless* gene in oocytes and granulosa cells. Our results indicated no apparent defect in fertility in oocyte-specific *Wntless* knockout mice. However, granulosa cell (GC) specific *Wntless* deletion mice were subfertile and recurred miscarriages. Further analysis found that GC-specific *Wntless* knockout mice had noticeably smaller corpus luteum (CL) in the ovaries than control mice, which is consistent with a significant reduction in luteal cell marker gene expression and a noticeable increase in apoptotic gene expression. Also, the deletion of *Wntless* in GCs led to a significant decrease in ovarian HCGR and β-Catenin protein levels. In conclusion, *Wntless* deficient oocytes had no discernible impact on mouse fertility. In contrast, the loss of *Wntless* in GCs caused subfertility and impaired CL formation due to reduced LHCGR and β-Catenin protein levels, triggering GC apoptosis.

## INTRODUCTION

After undergoing follicular growth, oocyte meiotic maturation, ovulation, and luteinization, the ovary ovulates mature oocytes for fertilization and forms the corpus luteum (CL) for implantation in female mammals. Additionally, the ovary produces steroid hormones such as estradiol and progesterone that are required for the development of female secondary sexual characteristics and the establishment of pregnancy [1]. These functions are regulated by the pituitary gonadotropins (FSH and LH) and ovarian-derived factors [1, 2]. For example, ovulation is triggered by the preovulatory surge of LH, which activates multiple gene expression and signaling pathways in the granulosa cells of preovulatory follicles. The expression levels of an inducible form of PGG/H synthase (PTGS2) and epidermal growth (EGF) like factors (AREG, BTC, EREG) are increased in response to the LH surge [3]. Previous studies have shown that EGF like factors, and its downstream MAPK signaling cascade, play pivotal roles in cumulus cell-oocyte complex expansion, oocyte maturation, and follicle rupture [4–7]. After ovulation, the ovary rapidly initiates terminal differentiation of the ovulated follicle into a CL through luteinization [8]. Luteinization is a process of rapid remodeling, growth, and differentiation. There are structural and genomics changes that lead to terminal differentiation of follicular cells into nondividing progesterone producing luteal cells [9]. *Lhcgr*, *Cyp11a1*, *Star*, and *Sfrp4* genes are highly expressed during this period [10]. When ovulated oocytes are not fertilized, the apoptotic system will eliminate CL, and many apoptotic genes (*p53*, *caspase 3*, *C-myc*) will have higher expression levels in the ovary.

One of the crucial factors shown to impact ovarian cell function is the WNT signaling pathway. The WNT signaling pathways, including WNT/β-Catenin; WNT/Ca^2+^; and WNT/Junkinase (Planar Cell Polarity) (WNT/ JNK(PCP)), have been shown to be vital to a variety of developmental processes. These processes include gastrulation movements, dorsoventral patterning, neuronal migration, maintenance of stem cell pluripotency, and disease states [11–14]. These pathways are all activated by WNT ligands, which bind to Frizzled (FZD) receptors and an array of co-receptors [15–17]. β-Catenin is an intracellular mediator of WNT/β-Catenin, or canonical WNT pathway, which is governed by the interaction of β-Catenin with other molecules to control diverse developmental processes such as cell fate specification, cell proliferation, and cellular differentiation [18, 19]. Gene knockout mouse models have provided some information about WNTs and WNT signaling in the ovary. For example, the deletion of β-Catenin in granulosa cells causes female infertility [20]. The WNT/β-Catenin pathway regulates FSH which in turn regulates steroidogenesis and LH-mediated ovulation and luteinization [10, 20]. In addition, the foundational study establishing the requirement of WNT signaling molecules for female ovarian function was performed by Vainio et al. [21]. This study found that *Wnt4* *null* mice have sex-reversed ovaries that express genes associated with testicular development, along with oocyte depletion [21]. Since then, a range of WNT ligands have been reported to be expressed in oocytes such as WNT2, 3, 5A, 7A or B, 10B and 11, and some expression of WNT2, WNT5A, and WNT11 also present in granulosa cells [22, 23]. Though the presence of many WNT proteins has been identified in the adult ovary of rodents, many questions remain regarding their mechanistic role in ovarian follicle development.

WNTLESS (also known as GPR177, or Evi), a regulator essential for intracellular WNT trafficking, is responsible for the secretion of WNT proteins from signaling cells [24, 25]. Loss of WNTLESS function impedes all WNT signals but has no effect on other signaling pathways [24, 25]. *Wntless* *null* mice die in the embryonic stage resulting from body axis establishment failure [26]. Subsequently, many *Wntless* conditional knockout mice were generated to study WNTLESS function in different tissue types. For example, Carpenter et al. showed that WNTLESS is not required for brain and pancreas development using Cre to remove *exon1*; Fu et al. deleted *Wntless* in WNT1 expressing cells, giving rise to mid/hindbrain and craniofacial defects; Zhu et al. found that WNTLESS controlled epithelial initiation of the fungiform placode by means of *Shh^Cre^* -mediated oral epithelial deletion of *Wntl*ess [27–29]. Further, the phenotype of *Wntless* deletion in WNT1-expressing cells resembles the double knockout of WNT1 and WNT3A as well as β-Catenin deletion [28]. It has been suggested that WNTLESS controls epithelial initiation of the fungiform placode through signaling via epithelial WNT ligands [29]. However, the complexity and overlapping expression of WNT signaling cascades have, to date, prevented researchers from elucidating their function in the ovary. To address this problem, we used *Wntless* conditional knockout (cKO) mice to investigate the role of total WNT proteins and WNT signaling in the ovary. Specifically, we generated *Wntless* cKO mice in which *Wntless* was explicitly disrupted in oocytes (*Wntless ^Flox/Flox^, Gdf9-Cre* and *Wntless ^Flox/—^, Ddx4-Cre*) and granulosa cells (*Wntless ^Flox/Flox^, Amhr2-Cre*). Given that many WNT proteins have been identified in the adult ovary of rodents, *Wntless* knockout mice are an optimal model to study the role of WNT signal pathways and proteins.

## RESULTS

### WNTLESS expression in the mouse ovary

**Figure 1:**
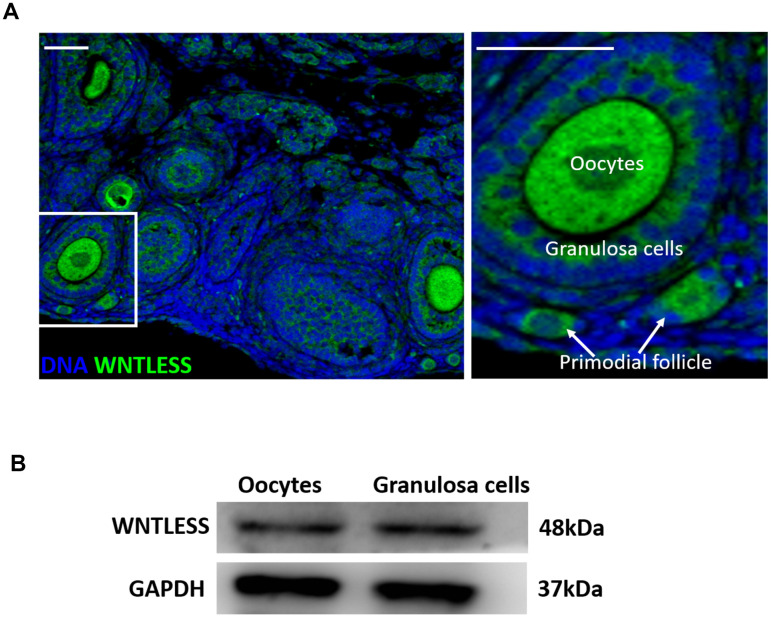
**WNTLESS expression in the ovary.** (**A**) The immunofluorescent staining of WNTLESS in the normal ovary. Green, WNTLESS; Blue, DNA. Scale bar, 100 μm. (**B**) The levels of WNTLESS protein in oocytes and granulosa cells are displayed by the western blot method. 200 oocytes and 10^6^ granulosa cells were used.

Our earlier studies suggest that WNTLESS is expressed ubiquitously in mouse tissues [30, 31]. In this study, we found that WNTLESS was highly expressed in follicles, including oocytes, granulosa cells, and primordial cells (Figure 1A). This result was confirmed by western blot, which showed the levels of WNTLESS protein in GV oocytes and granulosa cells (Figure 1B).

### Efficient and specific disruption of *Wntless*

**Figure 2:**
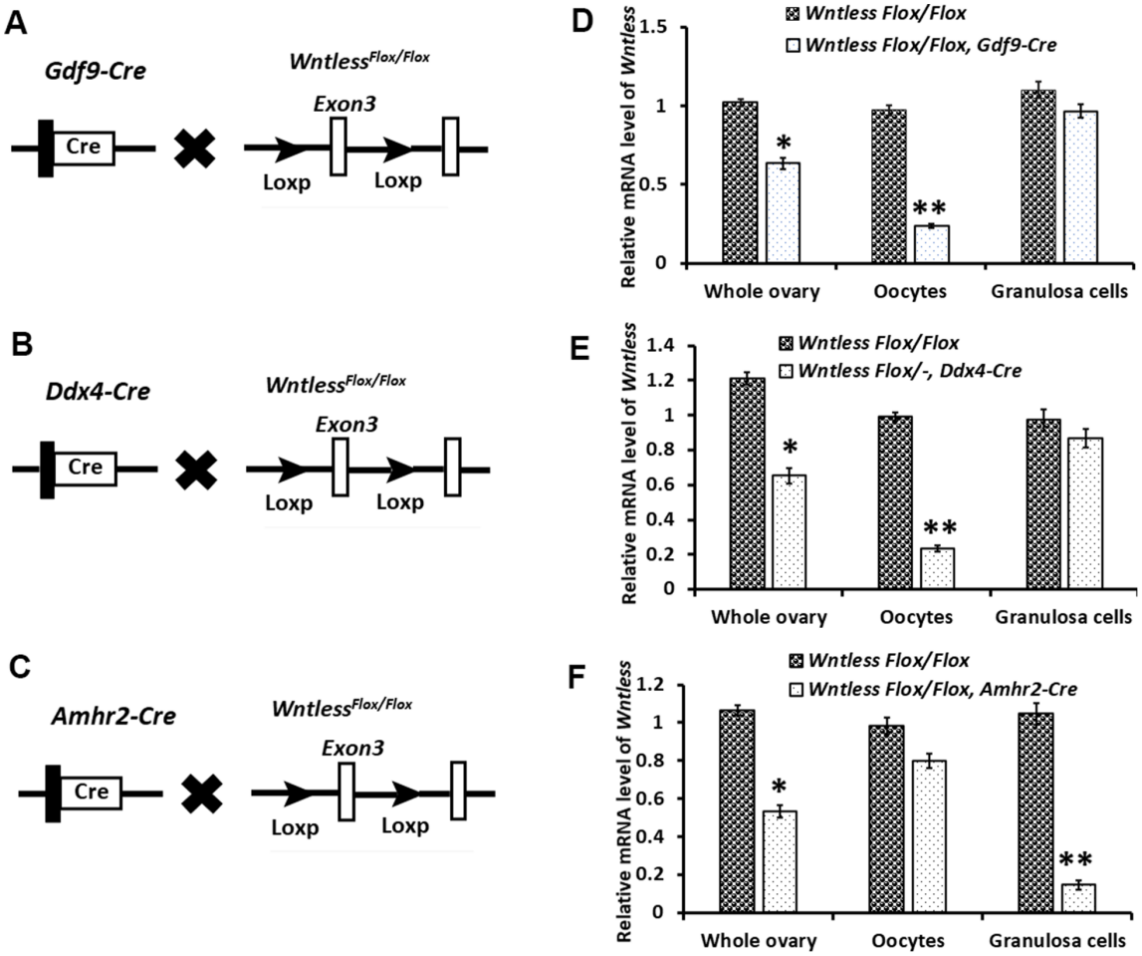
**Targeted disruption of the *Wntless* gene.** (**A–C**) The hybrid scheme used to develop *Wntless* knockout mice. Mice carrying a targeted *Wntless* allele (LoxP sites flank *Exon 3* of the *Wntless* allele) were crossed with *Gdf9-Cre* or *Ddx4-Cre* or *Amhr2-Cre* transgenic mice to delete *Wntless* selectively. The gene knockout was confirmed by PCR genotyping. The isolated genomic DNA from mouse tails was amplified with primer pairs specific for the wildtype (+) (~100 bp) and flox alleles (~200 bp) or different Cre bands (*Gdf9-Cre*: 326 bp, *Ddx4-Cre*: 240 bp and *Amhr2-Cre*: 156 bp). (**D–F**) qRT-PCR analysis showing the conditional loss of *Wntless* mRNA in total ovary, oocytes, and granulosa cells extracts of three *Wntless* knockout mice. *Gapdh* served as the internal control gene. The data are expressed as the mean ± SEM. *P<0.05, **P<0.01.

To assess the cell-type-specific function of WNTLESS during oogenesis, we generated mice in which the *Wntless* gene was disrupted explicitly in oocytes using *Gdf9-Cre* (Figure 2A) and *Ddx4-Cre* (Figure 2B) and in granulosa cells using *Amhr2-Cre* (Figure 2C)*.* *Wntless* deletion efficiency in oocytes and granulosa cells was assessed by detecting the *Wntless* mRNA levels in the whole ovary, oocytes, and granulosa cells, respectively. As expected, we observed a significant reduction in *Wntless* mRNA levels in the entire ovary and isolated oocytes from *Wntless^Flox/Flox^, Gdf9-Cre* (Figure 2D), and *Wntless^Flox/^*^-^*,* *Ddx4-Cre* mice (Figure 2E). Similarly, the *Wntless* mRNA levels were markedly decreased in both whole ovary and isolated granulosa cells from *Wntless^Flox/Flox^,* *Amhr2-Cre* (Figure 2F).

### *Wntless^Flox/Flox^,* *Amhr2-Cre* mice are subfertile despite apparently normal ovarian function and embryo development

**Figure 3:**
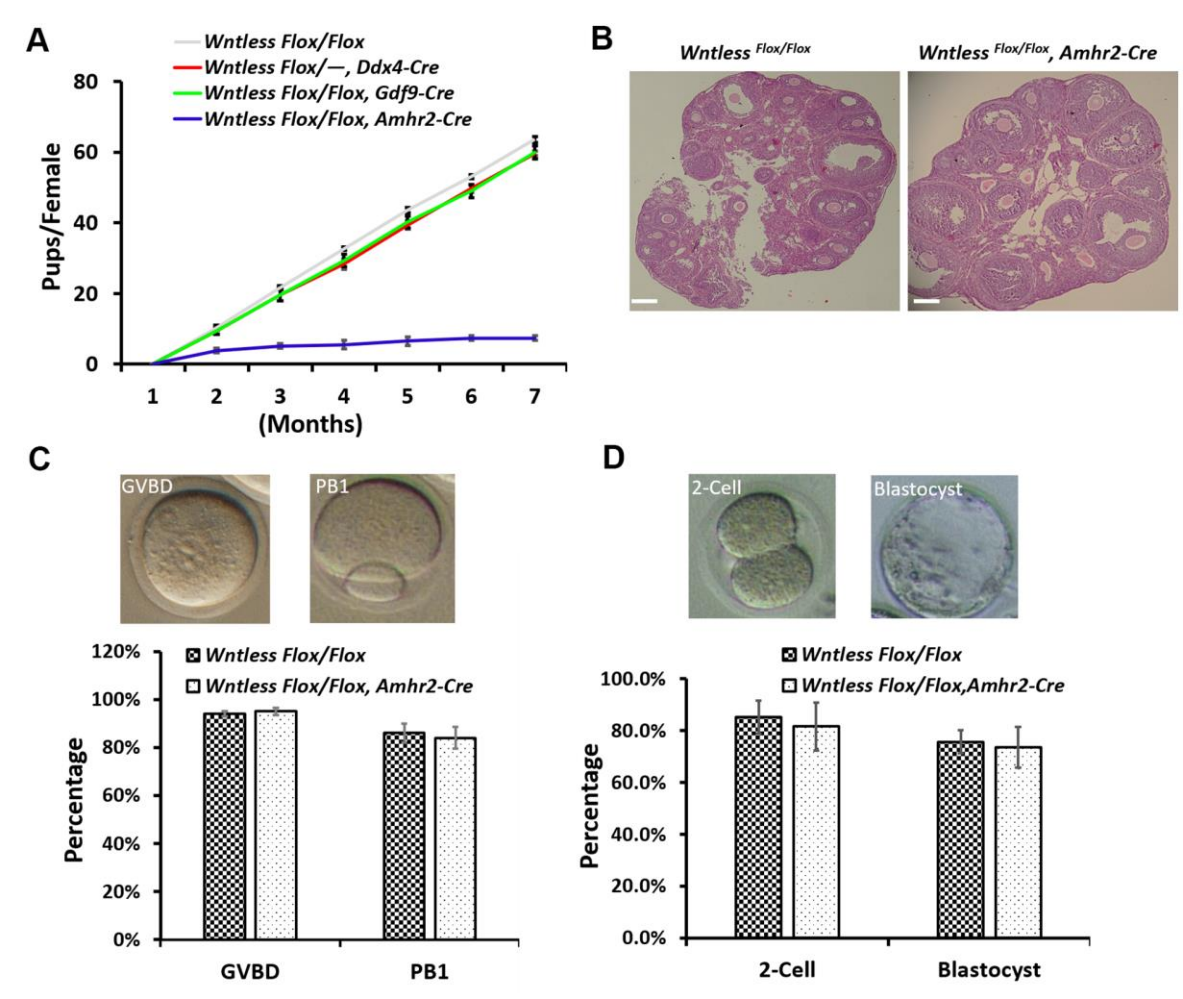
***Wntless^Flox/Flox^*, *Amhr2-Cre* females displayed subfertility with normal oocyte and embryonic development.** (**A**) Comparison of the accumulative number of pups per *Wntless^Flox/Flox^*, *Wntless^Flox/-^*, *Ddx4-Cre*, *Wntless^Flox/Flox^*, *Gdf9-Cre,* and *Wntless^Flox/Flox^*, *Amhr2-Cre* females (n = 6 for each group). (**B**) Histological ovarian images of *Wntless^Flox/Flox^* and *Wntless^Flox/Flox^*, *Amhr2-Cre* females. Scale bar = 100 μm. The ovaries were collected from 6-wk-old mice with a random cycle. (**C**) 345 and 400 GV oocytes from *Wntless^Flox/Flox^* and *Wntless^Flox/Flox^*, *Amhr2-Cre* females were matured in M16 medium, respectively. After maturing for 3 and 16 h, oocytes were counted, and the rates for GVBD and PB1 were calculated, respectively. Representative images of mouse oocytes at GVBD and PB1 stages are shown above the bar graph. (**D**) Zygotes collected from *Wntless* ^*Flox/Flox*^ (n=160) and *Wntless^Flox/Flox^*, *Amhr2-Cre* (n=193) females with an obvious vaginal plug after mating with wildtype male mice were cultured in KSOM medium for 1 and 3 days to calculate the rates of 2-cell and blastocyst, respectively. Representative images of mouse 2-cell and blastocyst are shown above the bar graph. Experiments were repeated a minimum of three times; the data are presented as mean ± SEM.

To study the effect of oocyte-specific and GC-specific deletion of *Wntless* on fertility, we conducted an animal breeding assay. Wildtype males were mated with 6-wk-old mutant and control females. The results indicated that oocyte-specific removal of *Wntless* had no significant impact on female mouse reproductive capacity (Figure 3A). However, the *Wntless^Flox/Flox^,* *Amhr2-Cre* females were significantly subfertile (Figure 3A), generating on average 1.2 offspring per female compared with 10.7 offspring in *Wntless^Flox/Flox^* females (Supplementary Figure 1). The subfertility may be due to either ovarian dysfunction, oocyte meiotic defect, or abnormal embryo development. Thus, to explore the reason for subfertility we investigated ovarian morphology, the rates of GVBD and PB1, the percentages of 2-cell and blastocyst in *Wntless^Flox/Flox^* (control) and *Wntless^Flox/Flox^,* *Amhr2-Cre* (mutant) females. Unfortunately, as shown in Figure 3B, 3C, and 3D, these parameters had no significant difference between the control and mutant females. In short, the subfertility initiated by the deletion of *Wntless* in GCs is not a consequence of ovarian dysfunction, aberrant oocytes, or embryo development.

### GC-specific *Wntless* knockout results in smaller CL and abortion in mice

Considering that the oocyte and embryonic development was normal in GC-specific *Wntless* knockout mice, we speculated that a problem might occur during pregnancy.

**Figure 4:**
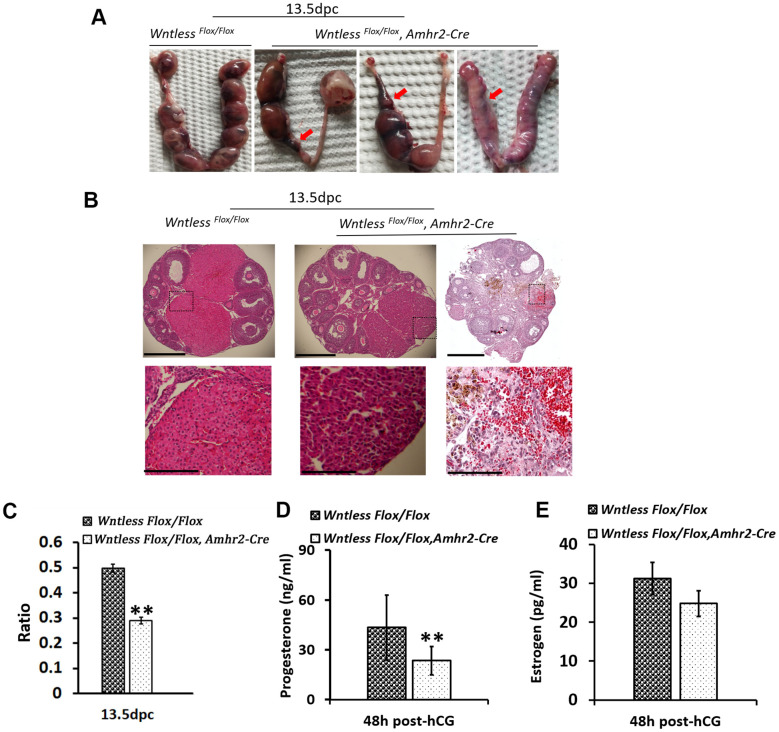
**Subfertility results from miscarriage and smaller CL in GC-specific *Wntless* knockout mice.** (**A**) Uteri collected from 13.5 dpc *Wntless ^Flox/Flox^* and *Wntless ^Flox/Flox^*, *Amhr2-Cre* mice. Red arrows indicate miscarriage locations. (**B**) Top row, representative images of one plane in ovaries, scale bar = 500 μm. The magnified images of a part of CL and hemorrhagic response are displayed in the second row, scale bar = 150 μm. (**C**) The area ratio of CL to the ovary for *Wntless ^Flox/Flox^* (n=5) and *Wntless ^Flox/Flox^*, *Amhr2-Cre* (n=6) mice at 13.5 dpc. (**D**) Serum progesterone levels in *Wntless ^Flox/Flox^* (n=5) and *Wntless ^Flox/Flox^*, *Amhr2-Cre* (n=6) mice at 13.5 dpc. (**E**) Serum estradiol levels in *Wntless ^Flox/Flox^* (n=5) and *Wntless ^Flox/Flox^/Amhr2-Cre* (n=6) mice at 13.5 dpc. In (**C**, **D**), **P<0.01.

To further evaluate the cause of subfertility in mutant females, uteri and ovaries in the control and mutant mice were collected at 13 days after the identification of a plug. Surprisingly, we found a high frequency of miscarriages in female mutants with few or no embryos in the uterus (Figure 4A). Further, some ovaries from mutant females had no apparent CL and appeared hemorrhagic (Figure 4B). Although some ovaries of knockout mice had a similar number of CLs with the control group, their CL size was smaller (Figure 4B and 4C) (*P<0.01*). Additionally, the luteal cells in the mutant mice exhibited more condensed nuclei, less cytoplasm, and gaps between cells (Figure 4B). CL is a temporary endocrine gland derived from the ovulated follicle and produces progesterone [32]. Progesterone is essential for the maintenance of pregnancy [32]. Thus, blood serum progesterone and estradiol levels were analyzed in the mutant and control mice at 13.5 dpc. As Figure 4E shows, estradiol levels had no apparent difference between the mutant and control mice. However, mutant mice had significantly lower levels of progesterone, reaching 28.3 ng/ml compared with 135.6 ng/ml in the control group (Figure 4D) (P<0.01). *Amhr2-Cre* is also present in the muscular layer of the uterus [33]. To rule out that the subfertility of mutant mice was caused by abnormal uterine morphology, histological analysis of uterus in mice at 8 and 24 h post-hCG treatment was conducted. As Supplementary Figure 2A, 2B show, no significant difference in uterine morphology was observed between mutant and control mice based on their appearance and HE staining. Additionally, mutant and control mice had a similar uterine and ovarian weight at 8 h post-hCG treatment (Supplementary Figure 2A).

Taken together, a lower level of progesterone caused by impaired CL may be the primary reason for abortion, which may cause subfertility in GC-specific *Wntless* knockout mice.

### Impaired CL formation and increased apoptotic genes in GC-specific *Wntless* deletion mice at 48 h post-hCG treatment

**Figure 5:**
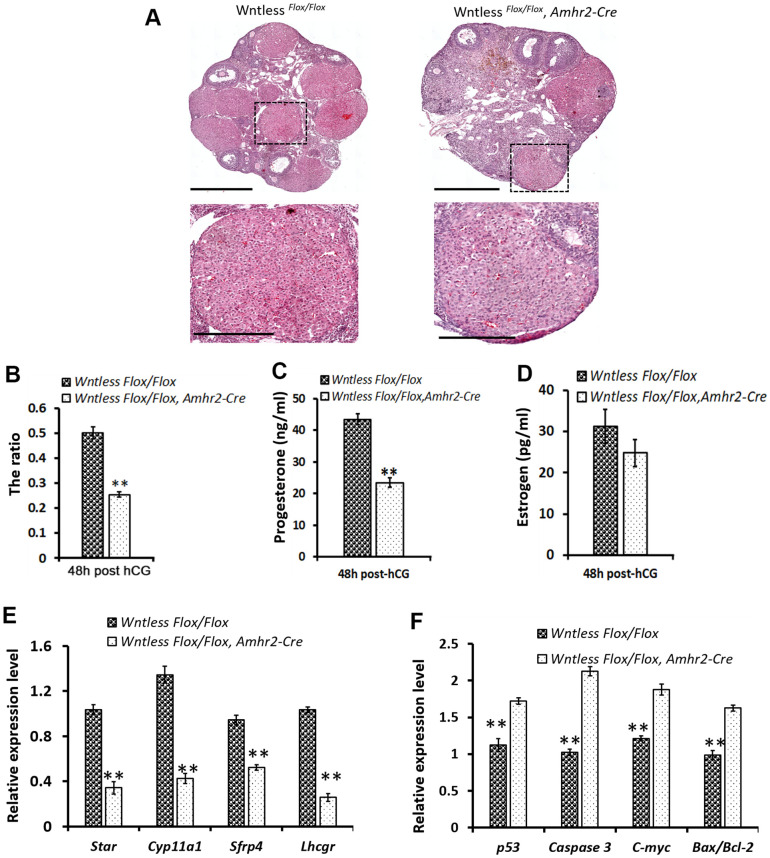
**Impaired CL formation in GC-specific *Wntless* knockout mice.** (**A**) Top row, representative images of control and mutant ovarian morphology at 48 h post-hCG treatment, scale bar = 500 μm. Second row, magnified images of CL, scale bar = 250 μm (**B**). The area ratio of CL to ovary for *Wntless ^Flox/Flox^* (n=6) and *Wntless ^Flox/Flox^*, *Amhr2-Cre* (n=6) mice at 48 h post-hCG treatment. Blood serum progesterone (**C**) and estradiol (**D**) levels in *Wntless ^Flox/Flox^* (n=6) and *Wntless ^Flox/Flox^*, *Amhr2-Cre* (n=6) mice at 48 h post-hCG treatment. Expression levels of luteal cell marker (**E**) and apoptotic genes (**F**), analyzed by qRT-PCR, in ovaries from 48 h post-hCG treated control and mutant mice. *Gapdh* served as the internal control gene. In B-F, **P<0.01, mean ± SEM. Experiments were replicated a minimum of 4 times.

CL starts to form after ovulation triggered by the preovulatory surge of LH [34]. To understand the cause of smaller CL in mutant mouse ovaries at 13.5 dpc, we assessed CL size in the mutant and control mice ovaries at 48 h post-hCG treatment. Histological analysis of the ovaries indicated few CL formations in the mutant ovaries, although CL's cell morphology had no apparent difference between the two groups (Figure 5A). Additionally, mutant ovaries appeared hemorrhagic (Figure 5A). Similar to ovaries at 13.5 dpc, the area occupied by CL in whole mutant ovaries at 48 h after hCG injection also had a significant decrease compared to control mice (*P<0.01*) (Figure 5B). In the control mice, blood serum progesterone levels reached 43.5±1.7 ng/ml at 48 h after hCG injection due to CL formation (Figure 5C).

However, serum progesterone levels were much lower in the hCG-treated mutant mice (Figure 5C) (23.5±1.6 ng/ml, *P<0.01*). Not surprisingly, serum estradiol levels had no significant difference between the mutant and control mice (Figure 5D). These findings suggest that GC-specific *Wntless* deletion causes fewer and smaller CL after ovulation. To explore the molecular changes underpinning impaired luteinization, qRT-PCR analysis of specific luteal cell markers and apoptotic genes was performed in ovaries from mutant and control mice at 48 h post-hCG treatment (Figure 5E, 5F). Result revealed that ovaries of GC-specific *Wntless* knockout mice had a significant decrease in mRNA expression of luteal cell marker genes (*Lhcgr*, *Sfrp4*, *Cyp11a1*, and *Star*) (Figure 5E) (*P<0.01*) and a visible increase in apoptotic mRNA expression (*p53*, *Caspase 3*, *C-myc* and *Bax/Bcl-2)* (Figure 5F) (*P<0.01*). In short, CL could not be efficiently formed after ovulation when *Wntless* was deleted in the granulosa cells of female mice.

### GC-specific deletion of *Wntless* has no impact on ovulation but leads to reduced β-Catenin protein level in the mouse ovary

**Figure 6:**
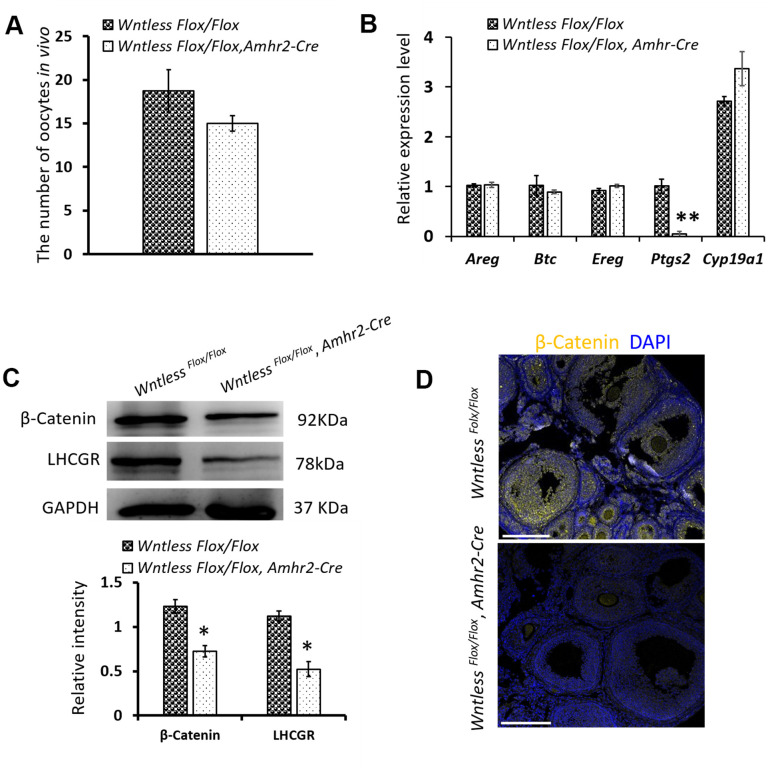
**The effects of *Wntless* deletion in granulosa cells on ovulation and the expression of β-Catenin and LHCGR.** (**A**) The number of ovulated oocytes in control (n=7) and mutant mice (n=6) after superovulation. (**B**) Relative mRNA levels of ovulation related genes in ovaries at 8 h post-hCG treatment. (**C**) The levels of β-Catenin and LHCGR in ovaries at 48 h post-hCG treatment measured by western blot. The β-Catenin and LHCGR levels were normalized to GAPDH. (**D**) Immunostaining of β-Catenin in the ovary at 8 h post-hCG treatment. Scale bar =100 μm. DNA, blue; β-Catenin, yellow. In (**A**–**C**), data are shown as the mean ± SEM. Experiments were replicated a minimum of 4 times.

Previous studies have shown that ovulation failure may also impair CL formation [1, 9]. To explore whether ovulation failure is a contributing factor in the decreased number of CLs, we assessed the number of ovulated oocytes *in vivo* in mutant and control mice after superovulation. As shown in Figure 6A, a similar number of oocytes were recovered from mutant and control mice. Consistent with this finding, the mRNA levels of ovulation controlling genes (*Areg*, *Btc*, *Ereg,* and *Cyp19a1*) in the ovaries of mutant and control mice had no significant difference at 8 h post-hCG treatment (Figure 6B). One exception was *Ptgs2*, where the mRNA levels were dramatically reduced in granulosa cells of *Wntless* deletion mice (*P<0.01*, Figure 6B). These results suggest that the reduced number of CL in *Wntless* mutant mice is not caused by ovulation failure.

WNTLESS is required for the secretion of various WNTs [24]. WNT/β-Catenin signaling plays a pivotal role in determining the fate of granulosa cells [35]. Thus, we hypothesized that *Wntless* knockout in granulosa cells might hamper the luteinization of granulosa cells by WNT/β-Catenin signaling. To confirm our hypothesis, the level of β-Catenin in the ovary was measured at 8 h post-hCG treatment by immunofluorescent staining and at 48 h post-hCG treatment by western blot. The result showed that β-Catenin levels were significantly compromised in the ovaries of mutant mice compared to control mice (*P<0.05*) (Figure 6C, 6D). In addition, LHCGR levels were markedly decreased in the mutant ovaries at 48 h post-hCG treatment (Figure 6C) (*P<0.05*).

Collectively, these results suggest that decreased β-Catenin levels may cause luteinization failure of granulosa cells in the ovaries of GC-specific *Wntless* knockout mice.

## DISCUSSION

WNTs are highly conserved signaling molecules that act through β-Catenin dependent and β-Catenin independent pathways to regulate essential processes of cellular growth and differentiation [36, 37]. In the adult ovary, specific WNTs are required for normal ovarian function and fertility. However, the broader physiological involvement of WNT signaling in the ovary remains largely unknown. Fortunately, recent studies have identified a novel WNT pathway component, WNTLESS, that promotes WNTs secretion from WNT-producing cells into extracellular milieu [24, 25]. WNTLESS, a seven-pass membrane protein, is evolutionarily and functionally conserved, and intriguingly, acts exclusively in WNT signal-sending cells [24, 25]. In this study we show, for the first time, the role of WNT signaling (both canonical and non-canonical) and all WNT proteins in the ovary through *Wntless* conditional knockout mice.

Previous studies have shown that WNT ligands are expressed in oocytes and granulosa cells [22, 23]. *Wnt7a* and *Wnt2* mutant mice do not appear to exhibit ovarian defects [6, 38] because WNTs may have distinct and overlapping roles during follicle growth, ovulation, and luteinization [39]. The deletion of *Wntless* in oocytes has little or no impact on female fertility (Figure 3A), which may also be masked by compensatory responses from granulosa cells in follicles where many WNT proteins are expressed [18, 40]. *Wnt4^flox/−^, Amhr2^cre /+^* females are subfertile, and some have tiny ovaries devoid of antral follicles [41]. Dysregulated WNT signaling may cause granulosa cell tumor development [18]. RNAi-mediated knockdown of *Wnt2* inhibits granulosa cell proliferation [42]. These studies indicate that WNT signaling is indispensable for granulosa cell proliferation and differentiation. In the present study, *Wntless^Flox/Flox^,* *Amhr2-Cre* mice were subfertile but had normal ovarian and uterine histology (Figure 3B and Supplemental Figure 2). In contrast to infertile phenotypes, healthy oocyte and embryonic development were observed in GC-specific *Wntless* knockout mice, similar to wildtype mice (Figure 3C, 3D). Therefore, we speculate that WNT signaling in granulosa cells may play a vital role that may not be compensated by signaling from the oocytes.

Granulosa cells of the follicle wall undergo a terminal differentiation process known as luteinization after ovulation. CL is one of the few endocrine glands whose function and survival are limited in scope and time [9]. Studies indicate that WNT 4 expression increases after hCG treatment and remains elevated in the CL during pregnancy [39]. Some *Wnt4^flox/−^,* *Amhr2^cre /+^* females are devoid of CL at 8 weeks of age [41]. In addition, WNTs act through binding FZD receptors [9]. *Fzd4* *null* ovaries exhibit impaired luteinization and reduced expression of genes known to be associated with luteinization [43]. As a result, it has been suggested that WNT4/FZD4 signaling is crucial for the regulation of luteal cell formation and function [9]. In the present study, the ovary of GC-specific *Wntless* knockout mice had a smaller size in CL despite normal follicular development and ovulation of fertilizable oocytes. Because the *Wntless^Flox/Flox^,* *Amhr2-Cre* phenotype is similar to that of *Fzd4* *null* mice, we speculate that WNT4/FZD4 signaling might be defective in CL of GC-specific *Wntless* knockout mice due to a lack of WNTs secretion.

The primary function of CL is to produce progesterone, which is required for the establishment and maintenance of pregnancy. Blood serum progesterone levels are much lower when luteinization of granulosa cells is inhibited in mice after hCG treatment [10]. Consistent with this finding, the progesterone levels were reduced in GC-specific *Wntless* knockout mice in this study (Figures 4D and 5C). Considering that most luteal cells originate from granulosa cells [9, 44], we speculate that the transformation of granulosa cells into luteal cells is impaired when all of the WNTs secretions are impeded in granulosa cells due to the deletion of *Wntless*. In brief, WNT signaling may play an essential role in the transformation of granulosa cells into luteal cells.

A key effector of the canonical WNT signaling pathway is β-Catenin, a protein that not only mediates cell-cell adhesion but also acts as a transcription factor. WNT/β-Catenin pathway components are expressed in ovarian granulosa cells [10]. In the presence of the WNT signal, β-Catenin dissociates from this complex and translocates to the nucleus, where it acts to modulate the transcriptional activity of a wide range of target genes [45]. Therefore, it is not surprising that β-Catenin can exert profound effects on granulosa cell proliferation, differentiation, and survival [18]. In the present study, we found that β-Catenin levels in the ovary are dramatically decreased when blocking all WNTs secretion from granulosa cells via *Wntless* deletion (Figure 6C, 6D). Given that misregulated WNT/β-Catenin signaling has a negative influence on cell fate determination [46, 47], the deletion of *Wntless* in mouse granulosa cells may affect the differentiation of granulosa cells into luteal cells through alterations of β-Catenin expression and distribution. It has been suggested that reduction in β-Catenin may affect the proliferation of granulosa cells via adjustment of Cyclin D2 as well as disrupt cell-cell communication critical for cell survival [20]. However, how β-Catenin affects the differentiation of granulosa cells that requires further study.

PTGS2 (also called COX-2) expression can be triggered by an LH surge in granulosa cells prior to ovulation. However, timing is species-specific [48]. It has been reported that PTGS2 can be induced by cytokines in inflammatory cells and is the target for the development of selective anti-inflammatory drugs [49, 50]. Early luteal development can be considered a kind of physiological injury with an inflammatory response [51]. PTGS2 may be one of the critical mediators of early CL formation [52, 53]. Consistent with this point, there was a dramatic decrease in *Ptgs2* mRNA in the ovary of GC-specific *Wnltess* deletion mice with impaired CL formation. During the transition from the ovary to the CL, a multitude of immune cells and cytokines infiltrate the preovulatory follicle and play a role in the regulation of early luteal development [54]. Immune cells also serve to abate an inflammatory response generated by the demise of luteal cells [55]. The luteolytic cascade appears similar to that of general acute inflammation. Because both show time-dependent infiltration by immune cells and drastic changes in vascular tonus and blood flow [56]. Thus, the hemorrhagic response observed in some GC-specific *Wntless* knockout mice ovaries at 24 h after hCG treatment and at 13.5 dpc may be related to an inflammatory response caused by dead and dying luteal cells. CL formation, maintenance, and luteolysis are related to many factors, multiple signaling pathways, and complicated cell processes [9, 44]. Microarray or RNA-seq analyses may be beneficial to expand the molecular landscape further and to understand how GC-specific *Wntless* deletion impacts the formation of CL and its maintenance.

In summary, WNTLESS in the ovary of GC- and oocyte- cKO mice has no apparent influence on oogenesis. Whereas, the ovaries of GC-specific *Wntless* deletion mice exhibit impaired luteinization, leading to miscarriage and low fecundity. Given that WNTLESS acts exclusively in WNT signal-sending cells [24, 25], the weak WNT/β-Catenin signal pathway may be the main factor contributing to the inefficient transformation of granulosa cells into luteal cells in GC-specific *Wntless* deletion mice.

## MATERIALS AND METHODS

### Mice

All animal studies were carried out in accordance with the protocols approved by the Institutional Animal Care and Use Committee at the Institute of Zoology (IOZ), Chinese Academy of Sciences (CAS). All mice were maintained in a C57BL/6;129 /SvEv mixed background and were housed in a controlled environment (12 h light/dark cycle, 22 ± 1° C, 60%–70% humidity) and fed ad libitum with standard chow. *Wntless ^Flox/—^, Ddx4-Cre*, *Wntless ^Flox/Flox^, Gdf9-Cre*, and *Wntless ^Flox/Flox^, Amhr2-Cre* mice were respectively generated by crossing *Wntless ^Flox/Flox^* with *Ddx4-Cre*, *Gdf9-Cre,* and *Amhr2-Cre* mice. Genotyping was performed on DNA samples prepared from 1 mm tail clippings obtained from 3-wk-old mice, as previously reported [30, 31].

### Fertility rate and embryonic implantation

For fertility testing, 6- to 8-wk-old *Wntless ^Flox/Flox^* (n = 7), *Wntless ^Flox/—^, Ddx4-Cre* (n = 7), *Wntless ^Flox/Flox^, Gdf9-Cre* (n = 7) and *Wntless ^Flox/Flox^, Amhr2-Cre* (n = 7) mice were separately mated with wildtype C57BL/6 males for 6 months in a 1:2 ratio. Litter sizes were assessed after birth. For implantation studies, female mice were placed with male mice and checked for a vaginal plug the following morning. Uteri and ovaries were collected at13 days after the identification of a plug (13.5 dpc).

### Superovulation, oocyte and embryo collection and culture

Superovulation was performed in mutant and control female mice. Mice received a single intraperitoneal injection of 10 IU of eCG per mouse (Ningbo Hormone Product Co. Ningbo) followed 48 h later by 10 IU hCG (Ningbo Hormone Product Co.). The number of oocytes at the Metaphase II stage (MII) was recorded in mice after 16 h hCG treatment. After 48 h eCG treatment, oocytes at the germinal vesicle (GV) stage were liberated from the ovary using 26-gauge needles and then collected in M2 medium. Subsequently, these GV oocytes were matured in an M16 medium for 2-2.5 and 16-17 h to calculate GVBD and first polar body (PB1) rates, respectively. The culture process took place in an incubator at 37° C with 5% ambient CO_2_.

To collect preimplantation embryos, superovulated *Wntless ^Flox/Flox^* and *Wntless ^Flox/Flox^, Amhr2-Cre* females were caged with 24-wk-old C57BL/6J wildtype males. Zygotes were collected from the ampullar region of the oviduct when a vaginal plug was found. The rates of 2-cell and blastocyst were calculated after these zygotes were cultured *in vitro* for 1 and 3 days in KSOM medium in a humidified 5% CO_2_ incubator at 37° C, respectively.

### Hematoxylin and eosin (H&E) and immunofluorescence staining

Uteri and ovaries were fixed overnight at 4-8° C in 4% PBS-buffered paraformaldehyde, and then stored in 70% ethanol and embedded in paraffin. Tissue sections (5 μm thick) were cut and mounted on glass slides. Sections were deparaffinized, rehydrated, and then stained with H&E. Areas of CL and whole ovary were calculated using Image J software. For immunofluorescence staining, experiments were conducted as previously reported [31]. Briefly, tissue sections were dewaxed, rehydrated, and treated with antigen retrieval. They then were blocked with 5% BSA for 1 h and incubated with primary antibodies against WNTLESS (1:100; Santa Cruz Biotechnology, mouse, sc-133635) or β-Catenin (1:50; Abcam, rabbit, 1247-1) overnight at 4° C. Sections were incubated with FITC conjugated secondary antibodies (1:200; Jackson ImmunoResearch, West Grove, PA, USA) for 1 h after washing three times with PBS. Following DNA staining with DAPI, slides were mounted on the cover glass and examined via immunofluorescence microscopy (Zeiss LSM 780).

### qRT-PCR analysis

Experiments were conducted as previously reported [31]. Briefly, RNA was isolated from testes using Trizol (TIANGEN, Beijing, China) according to manufacturer’s protocol. A PrimeScript RT Reagent Kit (Takara, Dalian, China) was used for reverse transcription of RNA samples and real-time quantitative PCR was performed with GoTaq qPCR Master Mix (A6001/2; Promega, Madison, WI, USA) according to] manufacturer’s protocols. The samples' CT values were normalized to corresponding *Gapdh* CT values, and relative expression levels were calculated using the 2^-ΔΔCT^ method. All primers for qRT-PCR are described in Supplementary Table 1.

### Serum analysis

Mice were anesthetized, and their blood samples were collected from their intraorbital venous plexus. Progesterone and estradiol levels were measured and analyzed using radioimmunity (Beijing North Institute of Biotechnology).

### Western blotting

Western blotting experiments were conducted as previously reported [31]. Briefly, 200 oocytes and granulosa cells were lysed in radioimmunoprecipitation assay lysis buffer containing protease inhibitor cocktail tablets (Roche, Basel, Switzerland). Protein concentrations were measured using the Bradford assay (Bio-Rad, Richmond, CA, USA). The proteins were electrophoresed under reducing conditions in 10% SDS-PAGE gels and transferred to polyvinylidene fluoride (PVDF) membranes. The blots were blocked in 5% BSA and incubated overnight at 4° C with anti-WNTLESS (1:800; Santa Cruz Biotechnology, mouse, sc-133635), anti-beta-catenin (1:500; Abcam, rabbit, 1247-1), anti-LHCGR (1:500, Abcam, mouse, ab204950) antibodies or anti-GAPDH (1:5000, Bioworld, mouse, MB001), followed by incubation with a secondary antibody (anti-mouse or rabbit horseradish peroxidase-coupled antibody, Jackson ImmunoResearch) for 1 h at room temperature. The membranes were scanned using an enhanced chemiluminescent detection system. The protein level was normalized to GAPDH abundance.

### Data analysis

All experiments were conducted with at least three replicates. The data were analyzed using Student’s *t-test* in Statistical Package for the Social Sciences (SPSS) 19.0 software (SPSS, Inc., Chicago, IL, USA). *P < 0.05 and **P < 0.01 values were considered statistically significant. The results are presented as the mean ± standard error (SEM).

## Supplementary Material

Supplementary Figures

Supplementary Table 1
