# Supplementary material for: Conditional deletion of Wntless in granulosa cells causes impaired corpora lutea formation and subfertility
Source: Aging (Albany NY). 2020 Dec 3;13(1):1001–16. doi: 10.18632/aging.202222 (PMC7835029; doi:10.18632/aging.202222)
Supplement: Supplementary Figures [file aging-13-202222-s001.pdf]

SUPPLEMENTARY FIGURES

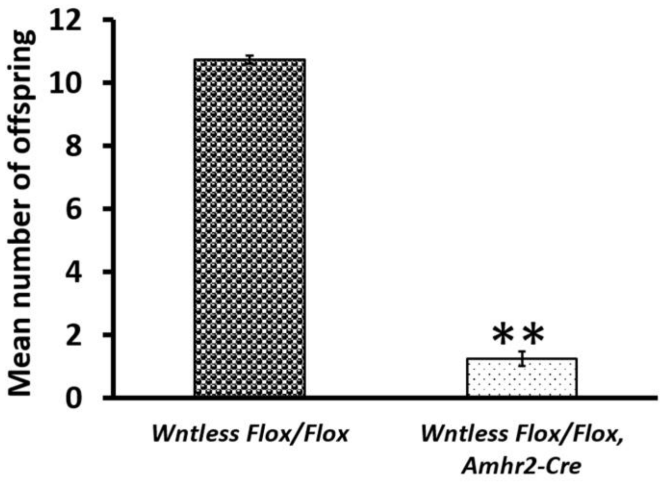

Supplementary Figure 1. Average litter size in the *Wntless* $^{Flox/Flox}$ and *Wntless* $^{Flox/Flox}$, *Amhr2-Cre* mice. ***P<0.01*.

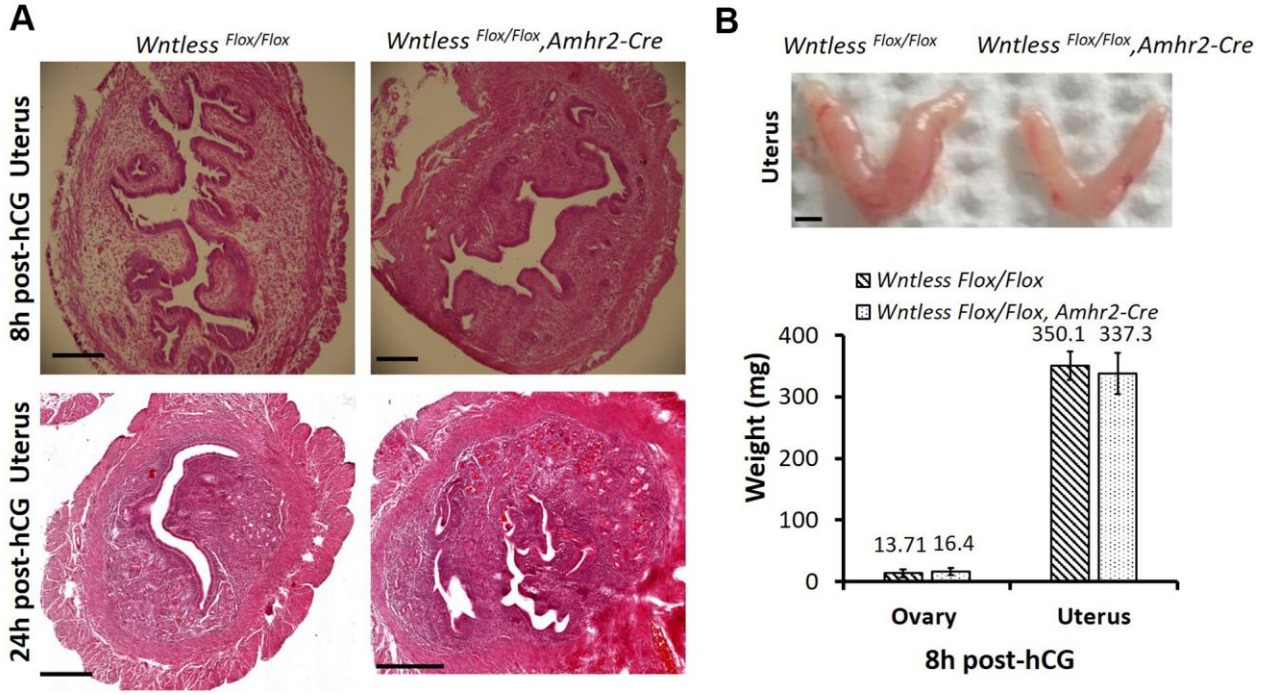

Supplementary Figure 2. The depletion of *Wntless* in mouse granulosa cells do not influence their uterine morphology and weight, and their ovarian appearance and weight. (**A**) HE staining of uterine morphology at 8 and 24 h post-hCG treatment. (**B**) Ovarian appearance (top) and weight (bottom) at 8 h post-hCG treatment.
