# Supplementary material for: Conditional deletion of Wntless in granulosa cells causes impaired corpora lutea formation and subfertility
Source: Aging (Albany NY). 2020 Dec 3;13(1):1001–16. doi: 10.18632/aging.202222 (PMC7835029; doi:10.18632/aging.202222)
Supplement: Supplementary Table 1 [file aging-13-202222-s002.pdf]

SUPPLEMENTARY TABLE

Supplementary Table 1. Primer sequence.

Gene name	Forward primers	Reverse primers
Gapdh	AGGTCGGTGTGAACGGAT	TGTAGACCATGTAGTTGA
Wntless	TGGGAAGCAGTCTAGCCTCC	GCAGCAAGCCAAGGTGATA
p53	CTCTGAGTATACCACCATCC	CACGAACCTCAAAGCTGTCC
Bax	TGCTGACGTGGACACGGACT	AGCAAAGTAGAAGAGGGCAACCA
Bcl-2	CGAGAAGAAGGGAGAATCACAGGA	AATCCGTAGGAATCCCAACCAGAG
C-myc	CTTCTCTCCGTCCTCGGATTCT	GAAGGTGATCCAGACTCTGACCTT
Caspase 3	CATACATGGGAGCAAGTCAG	CCATGAATGTCTCTCTGAGG
Star	ACCCTTGAGCACCTCAGCACT	CCCATCCACCCGGGACTGGAA
Sfrp4	CATCAAGCCCTGCAAGTCTG	TAAGGGTGGCTCCATCACAG
Cyp11a1	CAGACGCATCAAGCAGCAA	CTGGAGGCAGGTTGAGCAT
Areg	GGTCTTAGGCTCAGGCCATTA	CGCTTATGGTGGAAACCTCTC
Btc	AATTCTCCACTGTGTGGTAGCA	GGTTTTCACTTTCTGTCTAGGGG
Ereg	CTGCCTCTTGGGTCTTGACG	GCGGTACAGTTATCCTCGGATTC
Ptgs2	TTCAACACACTCTATCACTGGC	AGAAGCGTTTGCGGTACTCAT
Cyp19a1	TGTGTTGACCCTCATGAGACA	CTTGACGGATCGTTCATACTTTC
Lhcgr	ACTGGTGTGGTTTCAGGAATT	CCTAAGGAAGGCATAGCCCAT
